# 

**Published:** 1900-03-31

**Authors:** 


					THE HOSPITAL. "The standard of highest parityLancet. March 31st, 1900.
CADBURY'S CADBURY'S
OOCOA A
NO DRUGS OR ALKALIB8 USKD.
No. 705NVol. XXVII. GSfiSJfl Saturday,
March 31, 1900.
^Subscription Terms,] pRICE TWOPENCE.

				

